# Harnessing Health IT for Improved Cardiovascular Risk Management

**DOI:** 10.1371/journal.pmed.1000313

**Published:** 2010-08-24

**Authors:** Sue Wells, Robyn Whittaker, Enid Dorey, Chris Bullen

**Affiliations:** 1 Epidemiology & Biostatistics, University of Auckland, Auckland, New Zealand; 2 Clinical Trials Research Unit, University of Auckland, Auckland, New Zealand

## Abstract

Robyn Whittaker and colleagues argue that IT-based programs can improve cardiovascular disease management and patient empowerment, but must be accompanied by supportive social and political environments and active patient and clinician engagement.

**Summary Points**

- Cardiovascular disease (CVD) risk management through drug, dietary, and other interventions can significantly prevent or delay CVD events.
- Despite evidence for effectiveness, large gaps have been demonstrated between what is known and what is actually done in health care.
- Information technology (IT) has the potential to support clinicians to close these gaps throughout processes of care delivery.
- IT can be used to support identification of at-risk individuals, CVD risk assessment and management, care planning, patient self-management, and evaluation of improvements in care and health outcomes.
- To achieve the potential of IT-based programmes requires a supportive social and political environment, substantial organisational changes, and active patient and clinician engagement.

Cardiovascular disease (CVD) is one of the leading causes of death and disability worldwide, accounting for 16.7 million (29.2%) of total global deaths, 80% of them taking place in low- or middle-income countries [1]. While death is inevitable, premature death and loss of productive years of life from CVD is, for the most part, preventable. Much can be achieved by population-wide interventions that seek to lower cigarette and salt consumption, obesity prevalence, and blood cholesterol levels in the entire population [2]. At a personal health level, there is an abundance of evidence that reducing modifiable CVD risk factors (smoking, lipid fractions, blood pressure, diabetes) through drug, dietary, and other interventions can prevent or delay CVD events. Almost all adults could achieve a 50%–80% relative reduction in CVD risk if they took a combination of a lipid-lowering drug, a blood pressure lowering drug, and aspirin [3]. The higher the pre-treatment CVD risk, the greater the absolute benefit and therefore the greater the cost-effectiveness of treatment [4]. Therefore, many national guidelines now advocate identifying those at high risk of a future CVD event in the short-term and tailoring the intensity of management for individuals according to their baseline CVD risk.

## Is CVD Risk Reduction Happening in Practice?

Large gaps have been demonstrated between what is known to be effective and what is actually done in health care [5],[6]. Studies have shown that a formal CVD risk assessment to systematically identify high-risk patients is rarely conducted [7],[8], and audits of care indicate substantial under-use of effective CVD interventions by clinicians [9]–[12].

The reasons why clinicians do not follow patient management guidelines have been explored in some depth [13]–[15] and reflect a complex interplay of organisational, health system, provider, and patient factors. Barriers include lack of time, heavy workload and other competing demands, a lack of teamwork and organisation in the practice, paperwork, bureaucracy, and a lack of support to conduct preventive care [13]–[15]. Guidelines have been criticised for being too complex and focused on single diseases, whereas patients present with multiple problems. Even if clinicians have the ability, time, and resources to act on the evidence, for management to be effective the patient needs to understand and agree with the approach, be motivated to make changes, and be willing and able to adhere to a management plan.

## Can Technological Innovation Help to Close the CVD Evidence-Practice Gap?

There is growing interest in the potential of information technology (IT) to reduce human error and variation in patient care. A recent systematic review has investigated the impact of a wide range of health IT [16]. Three major benefits were found for quality of care (particularly in association with preventive care):

- increased adherence to guideline-based care;
- enhanced surveillance and monitoring; and
- decreased medication errors.

Table 1 shows where health IT could be used to support the process of CVD risk assessment and management in primary care practices. The focus is on tailoring interventions to specific patients based on recorded data and thus providing personalised prevention.

**Table 1:** Health IT Support for CVD Risk Assessment and Management Processes in Primary Care.

Process of Care	Examples of IT Support
Identification of eligible population	Electronic query of age/sex registers
Recalls and reminders	Automated patient reminder and recall letter plus laboratory forms
	Automated clinical reminder at time of consultation
CVD risk assessment	Electronic risk calculator
CVD risk communication	Electronic pictorial clinical and patient decision aids
CVD risk management	Computerised decision support tools integrated with the electronic patient medical record
CVD risk factor reduction	Internet and mobile phone-based behaviour change support programmes
Referral to speciality services; other clinical ordering	E-prescriptions and e-referrals with electronic communication back to primary care
	Telemedicine remote specialist consultations
Long-term chronic disease management/self-care	E-guidelines-based care plans and goal setting
	E-mail and mobile communications between visits
	Remote monitoring
	Patient access to their electronic health records
	Internet/mobile patient social support networks
Monitoring of processes, service performance, and quality improvement	Automated audit and feedback tools to clinicians and patients
Monitoring of outcomes	Electronic linkage of clinical profiles to health outcomes (e.g., mortality and hospitalisations)

Most of these technologies are predicated on

- the presence of an electronic medical record (EMR, defined as computer-based clinical data of an individual that are location-specific and kept by a single practice or office, health centre, or ambulatory clinic [17]); and
- the ability to register patients, query the database to identify patients of interest, and contact them.

Electronic queries to identify those eligible for CVD risk assessment and utilisation of simple rules-based automated reminder and recall systems have been increasingly used internationally since the late 1970s. Electronic calculators for estimates of CVD risk have been available since the early 1990s. However, only recently have more patient- and clinician-friendly CVD risk communication tools been developed (see Box 1 for an example).

**Box 1. Example of CVD Risk Communication Tool Currently Being Used in New Zealand**

Your Heart Forecast, a tool that graphically depicts absolute, relative, and long-term measures of CVD risk as well as a more accessible metric, your “cardiovascular age” or “heart age”. It also shows the benefit of risk factor reduction (http://www.yourheart.forecast.org.nz/) [35].

Computerised decision support systems (CDSSs) can rapidly and automatically convert the recommendations from evidence-based syntheses into guidance for clinicians, tailored for an individual patient's profile. Systematic reviews have found moderate evidence of CDSS effectiveness with respect to provider processes and performance, but the effects on patient outcomes are understudied and, where research has been conducted, inconsistent [18]–[20].

E-prescribing and e-referrals are becoming widespread in countries with a high degree of practice computerisation and Internet connectivity. The challenge is to add value to this system via more extensive two-way sharing of information. This could include notification of filled prescriptions and alerts in the practice system for un-filled prescriptions; notification of changes to care plans and medications by other providers; and missed appointments at other health services.

CDSSs can also form the basis of shared goal setting and the development of care plans for risk reduction. Instant electronic access and registration into self-management programmes removes barriers, such as having to attend another session, uses the “teachable moment” (i.e., being informed one is at high risk of an acute cardiac event) to enhance motivation, and can involve the primary care provider to motivate and tailor appropriately to the individual's risk profile. Internet-based programmes to support self-management and healthy behaviour change are proliferating. While there is limited evidence for effectiveness of IT-based self-management programmes, smoking cessation is one area that has been rigorously trialled. A recent meta-analysis of 22 randomised controlled trials found “… sufficient clinical evidence to support the use of Web- and computer-based smoking cessation programs for adult smokers” (RR 1.44, 90% confidence interval 1.27–1.64) [21]. Social networking Web sites are also being used formally and informally for such support. Patientslikeme.com is just one example of harnessing social networking to provide peer support for patients with the same conditions [22]. However, the social networking phenomenon does not easily lend itself to rigorous research of effectiveness.

Electronic patient portals or patient access to electronic health records can further empower patients to manage their disease and become active participants in their own health outcomes. However, there is limited research published on the effectiveness of these tools with respect to health outcomes, particularly for CVD.

Telehealth interventions include any telecommunications between a provider and patient between clinic visits, such as via phone, Internet, videoconference communication, and text messaging. A systematic review of telehealth interventions for the secondary prevention of coronary disease found significant differences in smoking, lipid levels, and systolic blood pressure in patients taking part in such interventions [23]. Mobile phones are likely to play an important role here in the future, allowing behaviour change support to be proactively sent directly to people wherever they are in formats from simple SMS/text messages to multimedia video messages. Such programmes can be tailored and personalised, and yet are eminently scalable to large diverse populations (see Box 2 for an example). Two recent systematic reviews of health care delivered via mobile phone found 26 studies in smoking cessation, diabetes care, physical activity, stress management, hypertension, and other conditions [24],[25]. These reviews reported significant behaviour change, with clinical and process improvements in the majority of studies.

**Box 2. Example of a Mobile Phone Programme Being Used in New Zealand and the United Kingdom**

Txt2quit is a free national text messaging smoking cessation programme in New Zealand that people can join by phone or online (http://www.quit.org.nz/txt2quit). It was implemented following a large randomised controlled trial which showed a doubling of short-term quit rates [36]. A similar study of this intervention, called txt2stop, is now being conducted in the UK.

Telemedicine has also taken the form of live remote specialist consultation (for patients in geographically isolated areas), or the transmission of diagnostic images/video/data for review by specialists. This also includes remote monitoring at home by devices that transmit data (e.g., ECG, blood pressure, weight, blood glucose) to a health care provider for interpretation and alterations to care. Home-based telemonitoring using Internet and mobile phone technology has been demonstrated to reduce hospital admissions for those with congestive heart failure and, if admitted, to reduce the length of stay [26],[27].

Finally, IT support is vital for monitoring processes and outcomes for clinical audit and research with pay-for-performance programmes such as the United Kingdom quality and outcomes framework based on the necessity of an “IT spine”. Health IT records are increasingly being used for CVD research, for example, to generate large risk prediction cohorts [28],[29].

## What Is Needed to Implement a Systematic Technology-Based Approach for CVD?

The benefits of implementing a systematic health IT approach could include improvements in the quality of care, reduced duplication, improving access to services in areas of growing demand and limited resources, and improved monitoring and evaluation. However, while some health IT systems implementations succeed, the majority are likely to fail [30]. The greater the personal and organisational change required by a system, the greater the risk of failure. Adverse effects of health IT implementation can include increased time for direct and indirect patient care [31], the loss of privacy and confidentiality, and possible changes to the doctor–patient relationship [32]. The time commitment involved with learning and implementing new systems may be substantial. The set-up costs of computerisation and implementation of health IT can be large and require ongoing investment. Furthermore, IT is a rapidly evolving science. By the time a large-scale project is completed, technology has often moved on [33].

Key elements that facilitate the development of a systematic technology-based approach are the availability of high quality national or international CVD guidelines, and local health IT specialists with the ability to develop reliable systems and the willingness and resources to maintain, sustain, and continue to innovate in response to consumer, clinician, and local health service needs. At a national level, policies that support prevention of CVD and long-term condition management need to be aligned with national IT policies that support the development of unified informatics standards for collecting health data, coding, messaging, and network infrastructure. While privacy and confidentiality concerns remain, there is a need for mature privacy statutes and ensuring consumers are aware of the potential uses of their health data.

Without clinical and health service buy-in, no IT solution will be successful. The solutions need to be effective, user-friendly, and integrated with all other systems, and ultimately must be efficient in terms of person-time. Berg describes this as a “… process of mutual transformation …” involving organisational change and allowing for change in the IT strategy arising out of the process of implementation [34].

In summary, CVD management requires partnerships between providers and patients, between staff within an institution, between service providers in the primary care setting, and between primary, secondary, and tertiary care services. For IT to achieve the potential to close the gap between evidence and practice and translate into positive patient outcomes there needs to be teamwork and a change in the way clinicians and services work together. This is particularly important for long-term health conditions such as CVD, where there is often unbounded need in the face of limited resources.
